# Calcinosis Cutis Universalis: A Review of Therapeutic Strategies and Surgical Management

**DOI:** 10.3390/jcm15030959

**Published:** 2026-01-25

**Authors:** Emma Giacometti, Jérôme Martineau, Ilias G. Petrou, Daniel F. Kalbermatten, Matteo Scampa

**Affiliations:** Department of Plastic, Reconstructive and Aesthetic Surgery, Geneva University Hospitals, Geneva University, 1205 Geneva, Switzerland

**Keywords:** calcinosis cutis universalis, connective tissue diseases, pharmacologic therapy, surgical management, treatment algorithm

## Abstract

**Background/Objectives**: Calcinosis cutis universalis is a rare and severe manifestation of dystrophic calcification, most associated with connective tissue diseases such as dermatomyositis, systemic sclerosis, and systemic lupus erythematosus. It is characterized by widespread deposition of calcium salts throughout the soft tissues, leading to pain, recurrent infections, restricted mobility, and significant impairment in daily functioning and quality of life. Management remains challenging due to the absence of standardized treatment guidelines with risks including delayed wound healing and recurrence. Adjunctive therapies may support symptom control in refractory cases. **Conclusions**: Management of calcinosis cutis universalis requires an individualized, multimodal strategy. Based on available evidence and expert opinion, a stepwise therapeutic decision-making algorithm integrating medical, minimally invasive, and surgical approaches is proposed to guide clinical practice and the variable efficacy of available therapies. This review aims to summarize current therapeutic strategies and to propose a pragmatic approach to clinical decision-making. **Methods**: A narrative review of the literature was conducted using PubMed and Google Scholar. The review focused primarily on calcinosis cutis universalis and severe or extensive forms of calcinosis cutis, with particular emphasis on surgical management and its integration with medical and minimally invasive treatments. **Results**: Pharmacological treatments—including bisphosphonates, calcium-channel blockers, tetracyclines, phosphate binders, probenecid, immunomodulatory agents, biologics, colchicine, sodium thiosulfate and JAK inhibitors—show heterogeneous and often partial efficacy, with more favorable responses in early or localized disease. Surgical interventions such as excision, curettage, CO_2_ laser ablation, and reconstructive procedures provide meaningful symptomatic relief in selected patients but are associated.

## 1. Introduction

**Figure 1:**
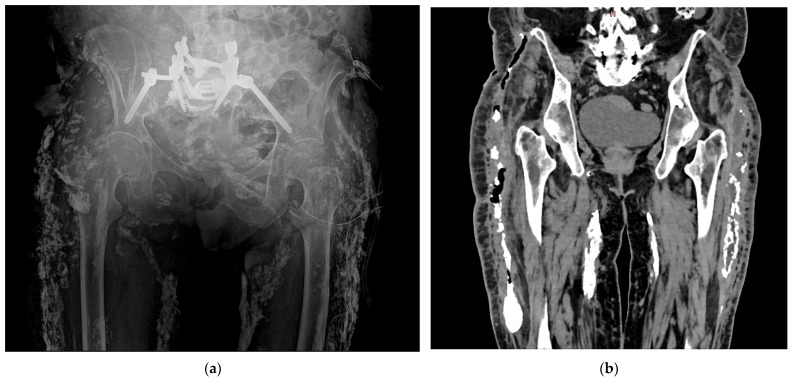
Radiologic assessment of a 65-year-old female patient with fever and known for calcinosis cutis universalis: (**a**) plain radiograph showing multiple calcifications along the hips; (**b**) further evaluation with CT scan demonstrating air adjacent to right sided calcified lesions, raising suspicion of secondary infection of calcinosis cutis universalis.

First described by Virchow in 1855, calcinosis cutis may present either in a localized form, known as calcinosis cutis circumscripta, or in a more generalized and disseminated pattern called calcinosis cutis universalis (Figure 1) [1]. Calcinosis cutis universalis is characterized by widespread deposition of calcium salts in subcutaneous tissues, fibrous muscle structures, fascial planes, and tendons [2]. There is currently no consensus on how to classify calcinosis cutis by size, but across the literature small lesions are typically defined as less than 1 cm and confined to superficial layers, while large or deep lesions are generally considered those with a diameter greater than 2 cm and/or extension into deeper tissues such as tendon or muscle [3].

Calcinosis cutis is classified into five subtypes based on the distinct underlying pathophysiological mechanisms: metastatic, dystrophic, idiopathic, iatrogenic and calciphylaxis [4]. Among patients with connective tissue diseases, the dystrophic subtype is the most frequent, occurring despite normal serum calcium and phosphate levels [5]. Calcinosis cutis universalis is a rare manifestation within this dystrophic category and is distinct from calciphylaxis, a severe and often fatal condition [6].

While calcinosis cutis universalis involves diffuse calcium deposits in soft tissues without vascular involvement, calciphylaxis is characterized by calcification of small- and medium-sized dermal arteries, leading to ischemia, painful skin ulcers, and necrosis. It primarily affects patients with end-stage renal disease and carries a high risk of sepsis and death [7].

The pathogenesis of dystrophic calcification involves cellular injury, due to connective tissue diseases or multiple traumas, inducing the release of phosphate-binding proteins from necrotic cells [8]. These proteins bind to free phosphate, facilitating the formation and deposition of insoluble calcium salts. In addition, the ongoing tissue damage provokes chronic inflammation and vascular hypoxia, worsening local tissue stress and calcific deposition. The resulting deposits are primarily composed of hydroxyapatite and amorphous calcium phosphate [4].

Multiple theories have been proposed to explain the development of dystrophic calcinosis, though its exact cause remains unclear. It is commonly believed to be linked to skin that is damaged, inflamed or necrotic [5]. Calcinosis cutis universalis typically arises secondary to connective tissue diseases such as dermatomyositis, systemic sclerosis, systemic lupus erythematosus, or Sjögren’s syndrome, all causing chronic tissue injury that can lead to pathological calcification [4]. Onset typically occurs within the first two decades of life, most commonly before the age of 20, and predominantly affects females [9].

Clinically, calcium deposits most often appear as subcutaneous nodules in areas exposed to repeated minor trauma, particularly the fingers, elbows, knees, and buttocks [10]. Symptoms can range from mild arthralgia to significant movement restriction, depending on the location and volume of calcium deposits [11]. Dystrophic calcifications are often painful, especially when located near joints or in cases of ulceration [12]. In contrast, muscle calcifications tend to be asymptomatic and are typically discovered incidentally through imaging studies [5]. Chronic ulceration may occur, leading to the drainage of calcific material, which can become secondarily infected or colonized by bacteria, resulting in persistent or recurrent infections [13].

The management of calcinosis cutis remains a considerable challenge and typically requires a multifaceted approach [4]. Most therapeutic recommendations stem from isolated case reports and small series, while randomized controlled trials are notably lacking. A wide range of pharmacologic agents has been proposed with the aim of reducing calcium deposition or preventing further progression of the disease [14]. Given the marked interindividual variability in treatment response, sequential and individualized therapeutic strategies are likely to offer the best chance of clinical benefit [8]. Alongside pharmaceutical therapies, supportive strategies, such as minimizing local trauma, smoking cessation, stress reduction, and avoidance of cold exposure, help improve peripheral blood flow and play an important role in disease management [4].

This narrative review outlines current treatments for calcinosis cutis, covering both surgical and nonsurgical options. Due to the limited literature on calcinosis universalis specifically, we included studies on all forms of calcinosis cutis to provide a comprehensive overview.

## 2. Methods

This narrative review was conducted through a literature search of PubMed and Google Scholar. Search terms included combinations of “calcinosis cutis universalis”, “connective tissue disease”, “treatment”, “surgery”, and “management”, using Boolean operators. Additional relevant publications were identified through manual screening of the reference lists of selected articles. Given the rarity of calcinosis cutis universalis and the predominance of case reports and small case series, a narrative approach was chosen to synthesize available evidence.

## 3. Treatments

### 3.1. Pharmacological Treatments

#### 3.1.1. Vitamin K Antagonists

Warfarin, a vitamin K antagonist, has been investigated for its potential to reduce calcinosis by inhibiting vitamin K-dependent gamma-carboxylation of matrix Gla protein, thereby preventing calcification [14]. Clinical benefit has been observed primarily in patients with small, recent lesions. Cukierman et al. reported improvement in two of three systemic sclerosis patients treated with low-dose warfarin (1 mg/day), and Yoshida & Torikai described radiographic improvement in fingertip calcinosis [15,16]. In contrast, Lassoued et al. found no benefit in patients with long-standing diffuse calcinosis [17]. Overall, warfarin has shown inconsistent and generally limited efficacy in calcinosis cutis, including severe and extensive forms [18]. Overall, low-dose warfarin appears safe and well tolerated, without bleeding complications, but efficacy is limited to early or localized disease [19] (see Table A1).

Evidence supporting the use of low-dose warfarin in calcinosis cutis is limited to isolated case reports and very small case series, with conflicting results, and therefore represents a low level of evidence.

#### 3.1.2. Bisphosphonates

Bisphosphonates target macrophages and reduce calcium turnover, potentially stabilizing or regressing calcified lesions [14]. Clinical outcomes are heterogeneous, with Rauch et al. reporting subjective and radiographic improvement with intravenous pamidronate, while Mukamel et al. observed complete resolution of calcinosis in juvenile dermatomyositis with alendronate [20,21]. Although generally well tolerated, bisphosphonates can cause osteonecrosis of the jaw, gastrointestinal symptoms, and electrolyte disturbances. Their use is most appropriate in refractory, painful, or functionally limiting cases [21].

Overall, evidence supporting the use of bisphosphonates in calcinosis cutis remains limited to retrospective observational studies—including a study of 7 patients—and isolated case reports, corresponding to a low level of evidence (see Table A2).

#### 3.1.3. Antibiotics

Tetracycline antibiotics, particularly minocycline, have been investigated in calcinosis for their therapeutic effects beyond antimicrobial activity, including calcium chelation, matrix metalloproteinase inhibition, and anti-inflammatory actions [22]. Robertson et al. reported clinical improvement in 8 of 9 patients with limited systemic sclerosis [23]. Nevertheless, efficacy has not been consistently reproduced [24]. Minocycline is generally well tolerated, with minor gastrointestinal side effects and occasional pigmentation of calcified lesions or teeth [23]. Importantly, long-term tetracycline treatment increases the risk of antibiotic resistance by selecting resistant strains and promoting resistant genes emergence, potentially persisting even after antibiotic discontinuation [25] (see Table A3).

In contrast, evidence for cephalosporins is minimal. A single case report described marked improvement of calcinosis cutis in a patient with morphea profunda after a 20-day course of intravenous ceftriaxone, possibly related to calcium binding and matrix metalloproteinase inhibition [26]. However, the role of ceftriaxone remains uncertain, and potential adverse effects include antibiotic resistance, biliary sludge and nephrolithiasis due to calcium-ceftriaxone precipitation [14] (see Table A4).

Evidence for antibiotic use in calcinosis cutis universalis is low, mainly extrapolated from low-level data from connective tissue disease–associated subtypes, such as systemic sclerosis and juvenile dermatomyositis, and isolated case reports.

#### 3.1.4. Calcium Channel Blockers

Diltiazem, a non-dihydropyridine calcium channel blocker, has been widely used for calcinosis, particularly in systemic sclerosis [24]. It is hypothesized to reduce intracellular calcium in macrophages and improve tissue oxygenation, thereby mitigating calcification [8]. Case reports describe partial to significant improvement [27,28,29]. A larger observational study on 47 patients with systemic sclerosis have failed to confirm consistent efficacy [30]. Effective doses typically range from 240 to 480 mg/day [27]. Hypotension and edema are the main adverse effects [19].

Evidence supporting the use of diltiazem in calcinosis cutis universalis is limited to observational data, primarily derived from case reports and small observational studies in connective tissue disease-associated calcinosis—most commonly systemic sclerosis with localized digital involvement—with inconsistent efficacy reported (see Table A5).

#### 3.1.5. Phosphate Binders

Aluminum hydroxide, an antacid with phosphate-binding properties, has been employed in dystrophic calcinosis, particularly in juvenile dermatomyositis, systemic lupus erythematosus, and idiopathic forms. By reducing intestinal phosphate absorption, it decreases calcium-phosphate precipitation [31,32]. The therapy is generally well tolerated, though caution is advised in renal insufficiency due to the risk of aluminum accumulation [14].

Available evidence remains limited to isolated case reports and small series, which describe regression of calcifications and improvement in mobility (see Table A6).

#### 3.1.6. Uricosuric Agents

Probenecid, a sulfonamide derivative with uricosuric properties, increases renal phosphate clearance by inhibiting tubular phosphate reabsorption, thereby lowering serum phosphorus and reducing the calcium–phosphorus product [33]. This mechanism is thought to both prevent new calcification and promote regression of existing deposits. Clinical experience, mainly from case reports, has shown promising results in juvenile dermatomyositis (JDM) and calcinosis universalis. Harel et al. reported dramatic improvement in JDM with normalization of phosphorus and reduction in calcified deposits, while additional reports described regression of subcutaneous and intermuscular calcinosis and functional recovery in calcinosis universalis [33,34]. Nakamura et al. further demonstrated efficacy in resistant JDM, with restoration of joint mobility after prolonged treatment [35]. The drug is generally well tolerated, with only occasional rash or diarrhea reported [19]. Given its safety and oral use, probenecid represents a useful adjunct treatment when standard therapies fail.

Overall, evidence for probenecid in calcinosis cutis universalis is limited to low-level observational data derived from isolated case reports, mainly in juvenile dermatomyositis and rare cases of calcinosis cutis universalis, with no controlled studies available (see Table A7).

#### 3.1.7. Intravenous Immunoglobulin (IVIG)

IVIG, an immunomodulatory preparation of pooled antibodies, modulates inflammation through multiple pathways, including macrophage suppression, cytokine regulation, and complement inhibition [36]. It has been used in refractory cases of dystrophic calcinosis cutis, calcinosis universalis, and ulcerated forms [37]. Clinical outcomes vary: marked regression and ulcer healing have been reported [36,37,38], while no benefit was observed in long-standing disease [39]. IVIG is generally well tolerated, with only mild side effects such as headache noted [38]. Despite high cost and variable efficacy, IVIG may benefit patients with severe, refractory, or steroid-resistant calcinosis, unresponsive to immunosuppressive regimens [38,40].

Evidence for intravenous immunoglobulin in calcinosis cutis universalis is also limited to low-level observational data from isolated case reports and small case series, mainly in dermatomyositis-associated calcinosis and occasional cases of calcinosis cutis universalis, with heterogeneous clinical outcomes reported (see Table A8).

#### 3.1.8. Intralesional Corticosteroids

Intralesional corticosteroid injections, most often with triamcinolone, have been explored in localized calcinosis cutis. Their proposed benefit derives from suppression of local inflammatory responses and inhibition of fibroblast activity [8]. Several reports describe regression of nodules, improved mobility, pain relief, and ulcer healing [41]. While intralesional therapy is generally well tolerated, rare paradoxical induction of calcinosis has been documented [42], probably due to local tissue damage [43]. This approach provides targeted anti-inflammatory benefit with minimal systemic exposure, making it a practical option for accessible and symptomatic lesions unresponsive to systemic treatment [44].

Evidence for intralesional corticosteroids in calcinosis cutis universalis is limited to low-level observational data from isolated case reports, mainly in localized disease (see Table A9).

#### 3.1.9. Calcium-Chelating Agents

Sodium thiosulfate (STS), an inorganic salt with calcium-chelating and antioxidant properties, has been widely studied in calcinosis cutis [8,22]. Topical STS has shown good effectiveness in case series, with reported improvement rates ranging from 68% to 78%, including cases of complete resolution [45,46]. Intralesional injections show partial benefit in small lesions: lesions measuring up to 0.2 cm respond best to topical treatment, whereas those up to 2 cm are more effectively managed with intradermal injections [47]. Intravenous STS has yielded disappointing outcomes in advanced or widespread calcinosis [48]. Tolerance is excellent with topical or localized administration, while systemic delivery carries more side effects, such as moderate gastrointestinal disturbance [48]. Overall, STS is best suited for mild-to-moderate localized lesions.

Evidence supporting the use of STS in calcinosis cutis universalis is derived from low- to moderate-level observational data, including case series and retrospective cohorts—primarily involving disease-associated connective tissue—with consistent benefit reported for topical and intralesional administration (see Table A10).

#### 3.1.10. Colchicine

Colchicine, an antigout drug with anti-inflammatory activity through microtubule inhibition [8], has been reported in isolated cases of calcinosis cutis. Colchicine has been reported in isolated case reports of calcinosis cutis, where it appears to reduce inflammation and pain and promote healing of ulcerated lesions, without consistent effects on calcified deposits. Evidence remains limited to observational studies [49,50,51] (see Table A11).

#### 3.1.11. Biologic Therapies

##### Anti-CD20 Monoclonal Antibody

Rituximab, an anti-CD20 monoclonal antibody, depletes B cells through antibody-dependent and complement-mediated cytotoxicity, therefore reducing autoantibody production. Its efficacy in calcinosis cutis is mixed: partial responses have been reported in JDM [52], whereas patients with systemic sclerosis or CREST syndrome experienced more significant regression of calcinosis [53,54]. Rituximab is generally well tolerated, though mild infections at calcinosis sites have been described [52]. Rituximab may be considered in refractory calcinosis associated with autoimmune connective tissue disease, particularly when other immunosuppressants fail.

Evidence for rituximab in calcinosis cutis universalis is limited to low-level observational data from small case series and isolated case reports, with variable efficacy across disease subtypes (see Table A12).

##### Anti-TNF-α Monoclonal Antibody

Infliximab, a TNF-α inhibitor, has been explored in refractory calcinosis associated with juvenile dermatomyositis and overlap syndromes, though evidence remains confined to isolated case reports and very small series [55,56]. Intravenously is the route of administration described in the medical literature and case reports for infliximab when used to treat calcinosis cutis, Riley et al. reported clinical benefit with systemic dosing at 6 mg/kg every four weeks, leading to improved muscle strength, pain relief, and regression of calcinosis [55]. More recently, Shiari et al. described an alternative approach using intralesional injections of 25 mg once weekly for six weeks, directly targeting calcified lesions [56]. Treatment was generally well tolerated in these reports, with infections rarely observed [55]. Despite promising clinical outcomes, infliximab use is limited by its high cost, an issue common to all biologic therapies and long-term treatment requirements. It may be especially valuable in aggressive or treatment-resistant cases.

Overall, available evidence supporting the use of infliximab in calcinosis cutis unviersalis remains limited to low-level observational data and small uncontrolled studies, with reported benefit mainly in refractory, autoimmune-associated cases (see Table A13).

#### 3.1.12. Immunomodulatory Agents

Thalidomide, an immunomodulatory drug that reduces TNF-α and IL-6 production, has been reported in a single case of severe, treatment-refractory JDM with calcinosis. Therapy led to resolution of systemic inflammation, pain, and fever, although calcific deposits persisted. The treatment was well tolerated in this case [57].

Evidence for thalidomide in calcinosis cutis universalis remains anecdotal, limited to one case report; nevertheless, despite important safety considerations, it may represent an option worth considering in highly selected, treatment-resistant cases (see Table A14).

#### 3.1.13. JAK Inhibitors

Janus kinase (JAK) inhibitors are targeted small-molecule immunomodulators that block JAK–STAT–mediated cytokine and interferon signaling. In dermatomyositis, this pathway is implicated in disease activity and may contribute to calcification through altered calcium handling [58]. Clinical experience with JAK inhibitors in calcinosis cutis remains limited but encouraging, primarily in refractory dermatomyositis (DM) and juvenile dermatomyositis (JDM).

Tofacitinib has been reported in two adult DM patients with extensive calcifications, showing rapid and sustained clinical response, stabilization or regression of lesions, absence of new calcifications over 28 weeks, and functional improvement [58]. Ruxolitinib has been described in a pediatric patient with severe MDA5-positive JDM, with resolution of skin lesions and disappearance of clinical signs of calcinosis in the affected region [59]. Baricitinib has also been reported in a child with refractory JDM, where progressive softening of calcium deposits and functional improvement occurred after its introduction within a combined therapeutic regimen [60].

Overall tolerance was acceptable in reported cases, with no major safety concerns, although all uses were off-label and involved very small patient numbers [58,59,60]. JAK inhibitors may represent a promising option for severe, refractory calcinosis associated with DM or JDM; however, evidence is limited to isolated case reports and very small case series, and their role in calcinosis cutis universalis remains unestablished (see Table A15).

### 3.2. Surgical Treatments

Excision of calcium deposits can be technically demanding, especially when complicated by lesion location, extension or underlying connective tissue disease [61]. Proper evaluation is essential, as calcinosis is occurring in up to 10% of patients with systemic sclerosis, 20% in adult patients with dermatomyositis and 10–40% of those with JDM, but is less frequent in systemic lupus erythematosus [62,63]. A comprehensive connective tissue disease workup is recommended, as these conditions represent the most common etiologies and directly influence prognosis, therapeutic choices, and surgical risk, given that underlying disease activity can impair wound healing and postoperative outcomes [64].

#### 3.2.1. Surgical Indications

Surgical intervention in calcinosis cutis is primarily indicated in cases of painful masses, recurrent infections, ulcerations, functional impairment, limited range of motion, discomfort, or cosmetic concerns [65]. Across connective tissue diseases, including systemic sclerosis, dermatomyositis, JDM, systemic lupus erythematosus, and idiopathic forms, surgery is often pursued when conservative medical treatments fail to provide relief. Although not curative, surgery is particularly justified when lesion-related symptoms significantly interfere with daily function or pose a risk of systemic infection. Reviews further confirm that well-selected surgical cases, especially those with localized deposits and manageable wound healing risk, often result in rapid pain relief, improved function, and reduced infection burden [15,66,67]. Surgical trauma may provoke additional inflammation and tissue injury, stimulating further calcium deposition, so excision should be reserved for select, symptomatic cases when alternatives are limited, given the risk of lesion worsening and high recurrence [32].

#### 3.2.2. Surgical Contraindications

Despite its benefits, surgical management is not without limitations. Several contraindications and precautions must be considered. Deep-seated calcifications involving vital structures, such as vessels, nerves or tendons, pose a higher surgical risk, particularly in functionally critical areas like the fingers [8]. Radical excision may compromise tissue viability or result in significant loss of function. Recalcification at previously treated sites is also a concern, potentially reducing the long-term success of surgery. In systemic diseases like scleroderma, microvascular pathology increases the risk of poor wound healing, as noted by Melone et al. [68], leading some clinicians to avoid surgery unless absolutely necessary. In general, small calcified deposits like plaques or nodules (<2 cm) or larger but localized lesions (deep deposits or calcinosis universalis) respond best to surgical intervention [14,22].

#### 3.2.3. Surgical Techniques

**Figure 2:**
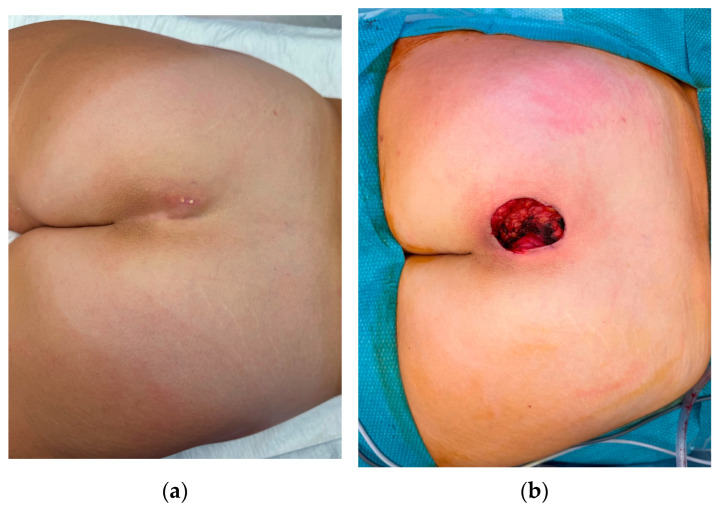
A 63-year-old female patient referred by her general practitioner for an extruding calcinosis cutis lesion: (**a**) preoperative view; (**b**) postoperative view after wide debridement. The defect was reconstructed using a rotation flap, and no recurrence was observed during follow-up (1 year postoperatively).

Several surgical techniques have been described for the management of calcinosis cutis, chosen according to lesion size, depth, and anatomical constraints. Excision and debridement remain the most common approach, offering rapid symptom relief for localized lesions, although the ability of this approach in halting disease progression remains uncertain (Figure 2a,b) [66,69,70]. However, when deposits are large, deep, or close to critical structures such as tendons, vessels, or nerves, the priority is to preserve both function, tissue integrity and avoid exposure [66]. In these situations, reconstructive techniques are essential. In more limited lesions, simple outpatient debridement can be sufficient [71]. Minimally invasive methods such as CO_2_ laser excision, curettage, or high-speed burr debridement have also been recommended to reduce trauma and promote faster healing in patients with compromised vascularity [14,22,63]. An additional refinement is the use of pulsed irrigation fluid, which facilitates the removal of calcific debris while minimizing dermal trauma and preserving microvascular circulation, thereby decreasing the risk of wound complications [69]. In JDM, techniques such as incision and drainage or broaching have been successfully employed to relieve pain and restore mobility, even when complete removal was not feasible [67,72]. Overall, the choice of surgical technique should balance radical removal of calcifications with preservation of function and tissue viability, tailoring the approach to lesion location and systemic disease context. Building on concepts described in other similar conditions, future research should investigate staged surgical excision combined with dermal substitutes and carefully planned reconstructive strategies using concealed donor sites, aiming to improve functional outcomes, reduce recurrence, and address the often-overlooked aesthetic burden of calcinosis cutis [73]. Emerging evidence suggests that combining surgery with adjunctive measures, such as negative pressure wound therapy, may further enhance postoperative healing, reduce infection risk, and minimize recurrence in complex or refractory cases [74].

#### 3.2.4. Complications

Surgical management of calcinosis cutis carries risk of complications that must be carefully considered before surgery, including local tissue damage, particularly involving digital neurovascular bundles, recurrence of calcific deposits and wound-related issues such as infection and hematoma formation [65,69]. Patients with widespread disease who are receiving long-term corticosteroids are at increased risk of wound healing issues, necessitating prolonged immobilization and elevation; staged excisions may be preferable [69]. Additional complications reported in the literature include skin necrosis and reduced joint mobility following wide excisions, particularly in systemic sclerosis patients with poor vascular supply [75]. When calcifications are deep and involve critical structures such as nerves, blood vessels, or tendons, excision may lead to functional impairment or significant tissue loss [66]. Importantly, recurrence of deposits is a well-recognized long-term issue, particularly in systemic diseases such as scleroderma or dermatomyositis, where ongoing disease activity promotes recalcification [14,63]. These complications can be mitigated by proper patient selection and meticulous surgical technique, including gentle handling of skin flaps, excision of devascularized edges, complete hemostasis with adequate drainage, tension-free closure, and protective splinting to reduce wound stress [69]. Despite these challenges, carefully planned surgery remains a valuable option, offering pain relief and functional improvement.

#### 3.2.5. Recurrence After Surgical Treatment

Although surgical excision is often effective for symptom relief, recurrence of calcium deposits remains a concern [14]. Mendelson et al. observed that while most patients experienced symptom relief following excision, minor recurrences were common, though they did not typically impair the overall functional gains achieved [69]. In Mendelson et al. series, most patients benefited from excision, but recurrence sometimes required reoperation, as in one case where new calcified nodules reappeared within three months, necessitating further surgery. Merlino et al. acknowledged the risk of recurrence in digital calcinosis but emphasized that careful surgical planning, particularly using flap reconstruction, may reduce this likelihood and obviate the need for further procedures [66]. In idiopathic cases, Guermazi et al. noted that local recurrence is not uncommon, yet surgery still provided significant symptom relief [76]. Different reviews corroborate that recurrence is a frequent concern, especially in systemic conditions like scleroderma or dermatomyositis, where ongoing disease activity may drive re-calcification [14,63,65]. Nonetheless, authors mention that recurrence risk does not preclude surgery or re-excision, and repeated debridement remain appropriate options when recurrence leads to renewed pain or functional impairment.

#### 3.2.6. Postoperative Outcomes

Postoperative outcomes following surgical excision of calcinosis cutis are generally favorable in terms of symptom control, though the long-term results remain variable. Mendelson et al. reported that most patients experienced significant symptomatic relief, with improved hand function and quality of life, even though surgery did not alter the underlying disease or the tendency to form new calcifications [69]. Merlino et al. similarly observed that radical debridement with vascularized flap reconstruction, particularly using Foucher’s kite flap, restored thumb mobility and grip strength with complete resolution of pain, although no data on recurrence was provided [66]. However, the literature agrees that surgery typically provides palliative rather than curative benefit, as recurrence is frequent and outcomes depend heavily on the disease context [14,63,65]. Nonetheless, even when relapse occurs, excision often translates into periods of pain-free function and improved daily activity, underscoring its value as a management option in selected patients.

### 3.3. Alternatives to Systemic Treatment and Surgical Excision

#### 3.3.1. Extracorporeal Shock-Wave Lithotripsy (ESWL)

ESWL a technique long used for kidney stones, has shown promising results in treating calcinosis cutis, primarily by reducing pain and improving quality of life [61]. According to Sultan-Bichat et al., ESWL significantly decreased pain scores and analgesic use in patients with refractory calcinosis, particularly in small (<2 cm), ulcerated, radiopaque lesions [61]. Grechin et al. observed long-term remission, reduced infections, and improved patient well-being over five years of treatment [77]. Nowaczyk et al., in a systematic review, confirmed ESWL’s potential but highlighted the limited evidence base, mostly from small, uncontrolled studies [70]. Current evidence supports ESWL as a safe, well-tolerated, non-invasive palliative option for pain control in treatment-resistant calcinosis cutis lesions ≤ 2 cm, particularly as an opioid-sparing strategy. However, larger controlled trials are needed to establish standardized protocols and confirm long-term efficacy.

#### 3.3.2. Laser CO_2_

CO_2_ laser therapy has been successfully used for superficial, localized calcinosis, offering surgical precision with less bleeding, faster healing, and good cosmetic outcomes [8,70]. Typically, a single session is sufficient to completely remove superficial and small calcifications up to 2 cm [19]. CO_2_ laser carries a lower risk of recurrence compared with conventional excision, specifically based on small lesions up to 2 cm [70]. Chamberlain and Walker reported pain relief and partial remission in CREST syndrome, while Bottomley et al. treated 21 digital lesions in systemic sclerosis with complete relief in 12, partial in 5, no improvement in 2, and only two recurrences, with healing in 4–10 weeks [78,79]. In systemic sclerosis, where wound healing is impaired, Daoussis et al. noted that CO_2_ laser is safer on small lesions than traditional excision, providing symptom control and functional improvement with fewer complications [54]. Compared with surgery, CO_2_ laser is less invasive, quicker, best for superficial symptomatic lesions, while excision is reserved for larger or deeper deposits requiring reconstruction.

## 4. Discussion

Most available evidence addressing the management of calcinosis cutis is derived from studies on calcinosis cutis in general, while data specifically focusing on calcinosis cutis universalis remain extremely limited. As a result, therapeutic strategies discussed in this review are largely extrapolated from broader calcinosis cutis populations and should be interpreted with caution when applied to the universalis subtype.

The therapeutic approach to calcinosis cutis relies primarily on stabilizing the underlying connective tissue disease, as persistent inflammation is a major driver of progression. Systemic therapies—including bisphosphonates, calcium-channel blockers, tetracyclines, phosphate binders, probenecid, IVIG, colchicine, and sodium thiosulfate—show heterogeneous and often partial efficacy, with responses influenced by lesion size, disease chronicity, and patient-specific factors. Biologic agents such as rituximab and infliximab have demonstrated encouraging results in selected refractory cases, particularly in autoimmune-associated calcinosis, but their use remains limited by cost, potential adverse effects, and low-quality evidence. Overall, medical therapy aims mainly at symptom control and slowing disease progression rather than achieving cure.

When conservative management fails or lesions cause pain, ulceration, infection, or functional impairment, surgical intervention becomes an important therapeutic option. Excision, curettage, CO_2_ laser ablation, and minimally invasive debridement can provide rapid symptomatic relief, although recurrence is common. Surgical risks—including delayed wound healing, neurovascular injury, and functional compromise—are particularly relevant in patients with connective tissue diseases or deep lesions. Surgery should therefore be reserved for carefully selected, symptomatic patients and ideally combined with optimized medical disease control.

### 4.1. Clinical Decision-Making and Therapeutic Sequencing

In the absence of validated guidelines, management of calcinosis cutis universalis must be pragmatic and individualized. Treatment selection should consider underlying disease activity, lesion size and depth, anatomical location, symptom burden, functional impact, and risk of complications. Early or inflammatory lesions may benefit modestly from medical or localized therapies, whereas long-standing, bulky, or mechanically limiting deposits often require procedural or surgical intervention.

**Figure 3:**
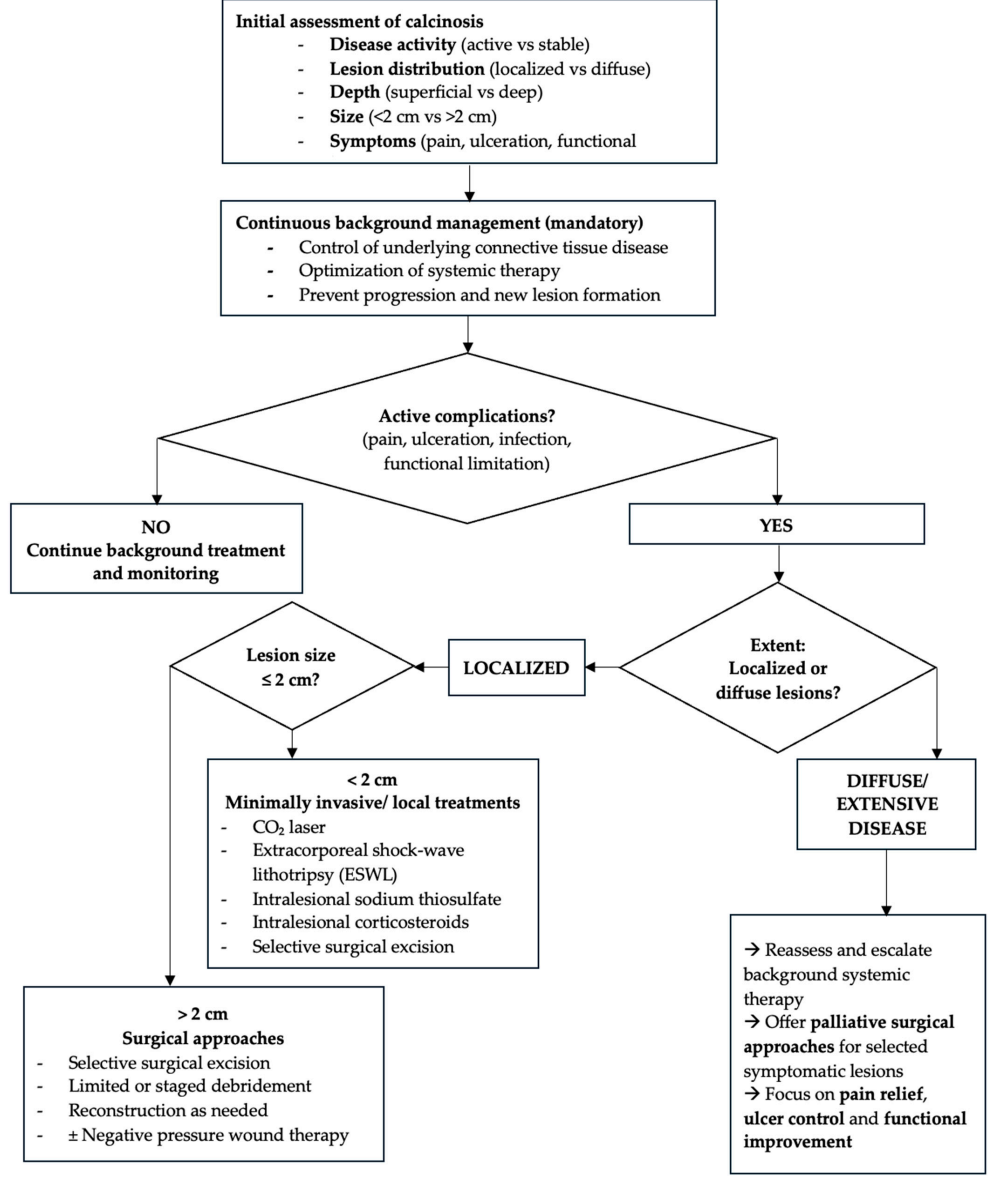
Expert-opinion-based therapeutic decision-making algorithm for calcinosis cutis. The algorithm integrates disease activity, lesion size, depth, distribution, and clinical impact to guide sequencing of medical, minimally invasive, and surgical therapies. This framework is proposed for clinical guidance in the absence of validated treatment guidelines.

To improve clinical applicability, we propose an expert-opinion–based therapeutic decision-making algorithm (Figure 3) that integrates medical optimization, minimally invasive techniques, and surgery in a stepwise manner. Although based on low-level evidence, this framework reflects current practice and provides practical guidance while acknowledging the predominantly palliative nature of available treatments.

### 4.2. Limitations of the Existing Evidence

The interpretation of therapeutic outcomes in calcinosis cutis universalis is substantially constrained by the quality of the available evidence. Most published data consist of single case reports or small, uncontrolled case series, frequently involving heterogeneous connective tissue diseases, variable disease durations, and mixed calcinosis subtypes. Outcome measures are inconsistently reported and often rely on subjective clinical improvement rather than standardized radiologic or functional endpoints.

Another important limitation is publication bias, as positive outcomes are more likely to be reported than treatment failures. Comparative studies between different medical or surgical strategies are lacking, and long-term follow-up data addressing recurrence, durability of response, and quality-of-life outcomes remain scarce. As a result, current therapeutic recommendations are largely based on expert opinion rather than high-level evidence, underscoring the urgent need for prospective registries and collaborative multicenter studies. Furthermore, most of the available data concern other subtypes of calcinosis cutis; therefore, the recommendations for calcinosis cutis universalis are largely extrapolated from treatments used for these other subtypes.

## 5. Conclusions

Calcinosis cutis remains challenging to manage, with treatment decisions largely individualized due to the absence of standardized guidelines. Conventional medical therapies may help stabilize the disease or reduce symptoms, but their benefits are often limited. Biologic agents show promising results but at the cost of potentially severe adverse effects. Surgery remains a valuable option for symptomatic, complicated or localized lesions. Further research is required to define clearer treatment algorithms and achieve more durable disease control.

## Data Availability

No new data were created or analyzed in this study. Data sharing is not applicable to this article.

## Appendix A

### Overview of Medical Treatments

**Table A1:** Warfarin.

Therapeutic class	Warfarin belongs to the vitamin K antagonists, which are commonly used as oral anticoagulants.
Mechanism of action	Warfarin may aid in treating calcinosis by inhibiting the vitamin K-dependent gamma-carboxylation of matrix Gla protein, which in its carboxylated form prevents calcification [8]. Elevated vitamin K levels observed in these patients normalize with warfarin use, potentially contributing to clinical improvement in small lesions [4]. Additionally, levels of calcium-binding amino acids like gamma-carboxyglutamic acid are increased in calcinosis and are linked to a warfarin-sensitive carboxylation pathway [19].
Specific indications	Warfarin is used in small calcified deposits and relatively new onset calcinosis [4,14,16].
Dosage and route of administration	In reported cases of calcinosis cutis associated with systemic sclerosis, warfarin was administered orally at a low dose of 1 mg per day over a period of one year. The low-dose approach aims to minimize anticoagulation risks [15,16,17].
Clinical efficacy	The clinical efficacy of low-dose warfarin in treating calcinosis cutis appears to be variable and may depend on lesion size and chronicity. Cukierman et al. reported that two out of three patients with systemic sclerosis and calcinosis cutis responded favorably to a one-year course of oral warfarin at 1 mg per day. The third patient—a 70-year-old woman with long-standing and extensive calcific lesions-did not respond to the same treatment. These findings suggest that low-dose warfarin may be more effective in patients with smaller and less chronic calcifications [16]. Similarly, Yoshida and Torikai described a case of fingertip calcinosis in a patient with CREST syndrome who showed marked radiographic improvement after low-dose warfarin monotherapy, further supporting its potential effectiveness in patients with localized and early-stage calcinosis [15]. In contrast, Lassoued et al. reported no clinical benefit in six patients with long-standing diffuse calcinosis due to dermatomyositis or scleroderma, all of whom received the same warfarin dosage for one year [17].
Side effects and tolerance	Low-dose warfarin appears to be well tolerated in patients treated for calcinosis, with minimal to no reported adverse effects. Across multiple studies, no patients experienced bleeding complications, clinical deterioration, or changes in baseline normal prothrombin time during treatment [19].
Role in therapeutic strategy	Systemic warfarin monotherapy has been shown to be effective for small calcified deposits, whereas no improvement has been observed in larger, longer-standing calcinosis cutis lesions [14,15,16].
Level of evidence	Very low (case reports and small case series only).
Type of calcinosis treated	Localized, small, early dystrophic calcinosis (often distal, e.g., fingertips) in systemic sclerosis/CREST.

**Table A2:** Bisphosphonates.

Therapeutic class	Three types of bisphosphonates have been reported in the literature relevant to this article: pamidronate, alendronate and etidronate.
Mechanism of action	Calcified lesions are often associated with the presence of macrophages and proinflammatory cytokines. Bisphosphonates target these immune cells by promoting their elimination and reducing the release of inflammatory mediators. They also help lower calcium metabolism, thereby limiting the calcium available for pathological deposition. For these reasons, bisphosphonates are believed to contribute to both the stabilization and partial regression of calcinosis [20].
Specific indications	Pamidronate, alendronate and etidronate are indicated in cases of calcinosis associated with autoimmune connective tissue diseases when lesions are extensive, painful, progressive, or functionally disabling, and especially when conventional treatments have failed [4,14,21,24].
Dosage and route of administration	Bisphosphonates are typically administered in combination with other therapies and most commonly by the oral route for alendronate and etidronate and intravenously for pamidronate. Reported dosages include 800 mg/day for etidronate, 10 mg/day for oral alendronate and 90 mg/week for pamidronate [20,21].
Clinical efficacy	The clinical efficacy of bisphosphonates in treating calcinosis varies depending on the patient profile and underlying condition. In Rauch et al., intravenous pamidronate provided subjective improvement in five out of seven patients and objective radiologic stabilization or regression in three, although one case of jaw osteonecrosis was reported [21]. Similarly, Martillotti et al. described a dramatic reduction in calcinosis and pain after three monthly doses of intravenous pamidronate in a child unresponsive to standard therapy [80]. Mukamel et al., demonstrated clear benefits from oral alendronate in juvenile dermatomyositis, including resolution of inflammation and full functional recovery within a year [20]. Balin et al., reported only one partial response among five patients, suggesting limited and inconsistent efficacy, reinforcing the idea that bisphosphonates are best suited for refractory cases [24]. These findings suggest that while bisphosphonates may not be universally effective, they can offer significant clinical benefits in selected patients, particularly those with refractory, painful, or functionally limiting calcinosis associated with autoimmune connective tissue diseases.
Side effects and tolerance	Bisphosphonates appear to be generally well tolerated, as reported in several case studies showing favorable clinical outcomes [20,21]. However, side effects can occur and include osteonecrosis of the jaw, fever, infusion site reaction, and transient decreases in calcium, phosphate, and magnesium levels [14]. Other reported adverse effects are gastrointestinal issues such as peptic disease, bone pain, and headache, as well as potential concerns about impaired growth in children. These risks highlight the need for cautious use, particularly in pediatric populations and during long-term treatment [19,80].
Advantages/disadvantages	Bisphosphonates may improve mobility and symptoms in calcinosis, with clinical improvement noted soon after treatment initiation [20].
Role in therapeutic strategy	Bisphosphonates play a role in the therapeutic strategy for calcinosis particularly in refractory cases. Alendronate and pamidronate have shown clinical benefit in patients with dermatomyositis or autoimmune connective tissue diseases when standard treatments failed [14,20,21].
Level of evidence	Low (retrospective case series and case reports/series).
Type of calcinosis treated	Dystrophic calcinosis cutis in systemic sclerosis, dermatomyositis, especially juvenile dermatomyositis.

**Table A3:** Minocycline.

Therapeutic class	Minocycline is a tetracycline antibiotic with anti-inflammatory properties.
Mechanism of action	Beyond its antimicrobial activity, minocycline exerts anti-inflammatory effects through inhibition of matrix metalloproteinases, suppression of neutrophil activity, and reduction in oxidative stress; calcium chelation may provide an additional contribution [22,23].
Specific indications	Minocycline was used to treat cutaneous calcinosis in patients with systemic sclerosis, particularly in cases complicated by pain, inflammation, or ulceration [23].
Dosage and route of administration	Minocycline was administered orally at a dose of 50–100 mg daily; either continuously or in cyclical regimens, 4–8 weeks on treatment followed by drug-free intervals [23,24].
Clinical efficacy	Clinical improvement with minocycline has been reported primarily in limited cutaneous systemic sclerosis. Robertson et al. observed improvement in 8 of 9 patients, including reduced pain, ulceration, and stabilization or reduction in calcinosis after a mean of 4.8 months [23]. In a larger observational cohort, 43.6% of patients with refractory calcinosis showed clinical improvement after repeated treatment courses, whereas limited efficacy was reported in another retrospective series (1 of 6 patients) [24].
Side effects and tolerance	Minocycline is generally well tolerated, with side effects such as nausea, dizziness, fatigue, and reversible blue-black pigmentation of calcified lesions [22,23].
Advantages/disadvantages	Advantages include oral administration, good tolerability, and effectiveness at low doses. Limitations include slow onset of action, need for prolonged or cyclical therapy, and potential cosmetic pigmentation of lesions [23].
Role in therapeutic strategy	Minocycline has shown potential as an adjunctive treatment for calcinosis cutis in limited systemic sclerosis, contributing to reduced lesion size and decreased inflammation or ulceration, particularly in cases unresponsive to other therapies [14].
Additional notes/comments	Some patients resumed minocycline after relapse, indicating that its effects may be reversible. Notably, all references to minocycline in the context of calcinosis are based on the case series by Robertson et al. [23], which remains the sole published report on its use in this setting. As a result, current evidence is limited and anecdotal, with no controlled studies available to confirm its efficacy.
Level of evidence	Low (case series and observational cohorts only)
Type of calcinosis treated	Dystrophic, localized, limited cutaneous systemic sclerosis.

**Table A4:** Ceftriaxone.

Therapeutic class	Ceftriaxone is a third-generation cephalosporin antibiotic.
Mechanism of action	Beyond its antibacterial properties, ceftriaxone acts as an anion capable of binding calcium to form insoluble complexes. It also inhibits several matrix metalloproteinases, which are involved in tissue remodeling and inflammation. These non-antimicrobial actions may help reduce both inflammation and calcium deposition [14].
Specific indications	Used in localized and inflammatory forms of calcinosis cutis, particularly when refractory to standard treatment [26].
Dosage and route of administration	In the reported case, ceftriaxone was administered intravenously at 2 g/day for 20 days [26].
Clinical efficacy	Reiter et al. described a 16-year-old patient with morphea profunda (localized scleroderma spectrum) and calcinosis cutis who experienced a marked reduction in calcified lesions within weeks, suggesting good short-term efficacy [26].
Side effects and tolerance	Known adverse effects include biliary sludge (pseudolithiasis) and nephrolithiasis, due to precipitation of calcium-ceftriaxone complexes in the biliary or renal systems.
Role in therapeutic strategy	Ceftriaxone may be considered as an alternative treatment for localized or inflammatory calcinosis, particularly in cases unresponsive to conventional therapy.
Level of evidence	Very low (single case report only).
Type of calcinosis treated	Localized dystrophic calcification/calcinosis cutis (in morphea profunda/localized scleroderma).

**Table A5:** Diltiazem.

Therapeutic class	Diltiazem belongs to the therapeutic class of non-dihydropyridine calcium channel blockers.
Mechanism of action	Diltiazem is hypothesized to reduce calcinosis by modulating intracellular calcium concentrations, especially in macrophages, potentially inhibiting the formation of calcium deposits [78]. It may also improve local tissue oxygenation and alleviate vascular insufficiency, thus reducing the tissue damage-calcification cycle [27,63].
Specific indications	Diltiazem is the most frequently used agent due to its widespread use in systemic sclerosis in some cases of dermatomyositis [8,63]. Balin et al. recommend diltiazem as first line treatment, often associated with surgical excision [24]. Most cases reports used diltiazem on calcinosis localized in hands and fingers [27,28,29,30].
Dosage and route of administration	Diltiazem is administered orally. Efficacy is typically observed only at high doses, ranging from 240 to 480 mg/day [8,27,81]. Lower doses, such as 180 mg/day, appear ineffective [14].
Clinical efficacy	Evidence remains inconclusive. While some case reports suggest partial to significant improvement [27,28,29], larger studies—such as the one conducted by Vayssairat et al.—have demonstrated limited or inconsistent efficacy [30]. The therapeutic response appears to be highly variable and may depend on factors such as dosage, duration, and individual disease characteristics.
Side effects and tolerance	Diltiazem is generally well tolerated. The main reported side effects include hypotension and peripheral edema [19]. Two case reports described patients who were unable to tolerate diltiazem, necessitating a change in treatment [27,82].
Role in therapeutic strategy	Its role is unclear and not standardized. Bienvenu lists it as a commonly used first-line systemic agent, often with colchicine [8]. However, Reiter et al. consider it a second-line or adjunctive option for refractory cases [14]. Vayssairat et al. reported no consistent benefit in SSc patients, suggesting its use is empirical rather than evidence-based [30]. According to Balin et al., diltiazem was the most frequently used and effective medical therapy for calcinosis cutis, supporting its role as a first-line option in patients for whom surgical excision is not feasible [24].
Additional notes/comments	Despite its frequent use, the long-term effect of diltiazem remains debated [4,8]. Differentiating between spontaneous regression and treatment effect is difficult. While a curative effect hasn’t been shown, a preventive effect in digital calcinosis, especially in systemic sclerosis, cannot be ruled out [30]. All case reports on diltiazem focus on lesions in the fingers and hands. Only Khudadah et al. [82] reported its use in generalized systemic calcinosis. Further controlled studies are needed to evaluate its broader efficacy.
Level of evidence	Low (case reports + small case series + one negative observational study).
Type of calcinosis treated	Localized dystrophic calcinosis, Digital calcinosis (hands/fingers), Mainly in systemic sclerosis/CREST. There is only 1 case report on generalized calcinosis [82].

**Table A6:** Aluminum hydroxide.

Therapeutic class	Aluminum hydroxide is in the antacid class of drugs, it binds phosphorus and reduces the intestinal absorption of phosphorus [4].
Mechanism of action	Phosphate is known to play a crucial role in the development of calcinosis cutis, as ectopic calcified masses have been shown to contain hydroxyapatite and amorphous calcium phosphate [14]. Aluminum hydroxide interacts with phosphate by forming insoluble aluminum phosphate salts, thereby reducing intestinal phosphate absorption [8,14].
Specific indications	Aluminum hydroxide has been used as an adjunctive treatment in calcinosis cutis, particularly in cases of dystrophic calcinosis associated with connective tissue diseases such as juvenile dermatomyositis, systemic lupus erythematosus (SLE), and idiopathic forms. It is especially indicated in calcinosis universalis, where calcium deposits are widespread in subcutaneous and muscular tissues, leading to significant morbidity [31,32].
Dosage and route of administration	Its safety has been demonstrated at the doses typically used between 1.8 and 2.4 g per day orally [31]. Additionally, topical administration such as 10 mL of aluminum hydroxide gel four times daily has also been employed with reported clinical improvement [32].
Clinical efficacy	Several case reports have demonstrated regression of calcified deposits following oral aluminum hydroxide therapy. Park et al. noted partial improvement–softening and reduction in calcification-in a patient with SLE with oral aluminum hydroxide, followed by surgical excision for optimal results [31]. Jatana et al. reported lesion softening and size reduction in idiopathic calcinosis cutis using topical aluminum hydroxide gel [32]. These cases-primarily in the context of dermatomyositis and lupus-illustrate the therapeutic potential of aluminum hydroxide, especially for reducing calcified deposits in both pediatric and adult forms of calcinosis cutis.
Side effects and tolerance	Aluminum hydroxide therapy has generally been well tolerated. However, caution is advised in patients with renal insufficiency, particularly in SLE, as impaired excretion may lead to aluminum accumulation and potential complications such as osteomalacia, myopathy, or neurotoxicity [19]. While concerns have been raised about the risk of reduced bone mineralization during prolonged use, this has not been observed in reported cases to date; nevertheless, monitoring of bone health is recommended during treatment [14].
Role in therapeutic strategy	Aluminum hydroxide is not considered a first-line treatment for calcinosis cutis but is used as a supportive or second-line option, particularly in refractory or extensive cases. Its role is primarily based on case reports and small case series, especially in patients with dermatomyositis, juvenile dermatomyositis, systemic lupus erythematosus, and idiopathic calcinosis cutis [31,32]. It is typically used as a monotherapy or in combination with other immunosuppressive or anti-inflammatory treatments (e.g., corticosteroids, hydroxychloroquine), when phosphate reduction is needed alongside disease control [31]. However, clinical reports indicate that while aluminum hydroxide therapy often leads to partial improvement in symptoms and a reduction in calcified lesions, full resolution is uncommon [8].
Level of evidence	Very low (single case reports only).
Type of calcinosis treated	Localized but large dystrophic calcinosis (SLE) and idiopathic diffuse/extensive case often cited as universalis-like.

**Table A7:** Probenecid.

Therapeutic class	Probenecid is a sulfonamide derivative classified as a uricosuric agent known for inhibiting uric acid reabsorption in the proximal tubule [19,32].
Mechanism of action	Probenecid increases renal phosphate clearance by inhibiting tubular phosphate reabsorption. This leads to lower serum phosphorus levels, which reduces the calcium-phosphorus product and may thereby limit calcium deposition in tissues. This mechanism is believed to both prevent further calcification and promote the regression of existing deposits [4,19,33].
Specific indications	Probenecid has been used for extensive calcinosis in juvenile dermatomyositis (JDM), particularly in patients with elevated serum phosphate and increased renal phosphate reabsorption [14,33].
Dosage and route of administration	Administered orally in doses ranging from 250 to 2000 mg/day [19].
Clinical efficacy	Several case reports highlight the clinical efficacy of probenecid in treating calcinosis. Harel et al. described a case of juvenile dermatomyositis with extensive calcinosis that showed dramatic clinical and radiological improvement, including pain relief, reduction in calcified deposits, and improved bone mineral content [33]. Similarly, Skuterud et al. reported marked regression of subcutaneous and intermuscular calcinosis in a 9-year-old girl [34]. Eddy et al. documented major functional and radiographic improvement in a 19-year-old patient with calcinosis universalis after seven months of therapy [83]. Lastly, Nakamura et al. reported a case of juvenile dermatomyositis with calcinosis resistant to multiple therapies, which improved remarkably following 17 months of probenecid, including normalization of serum phosphorus and restored joint mobility [35].
Side effects and tolerance	Side effects of probenecid may include rash and diarrhea, though this has been reported by only one source [19]. Across reported cases, probenecid was consistently well tolerated. Harel et al., Skuterud et al., Eddy et al., and Nakamura et al. reported no significant side effects during treatment, indicating a favorable safety profile in managing calcinosis.
Advantages/disadvantages	Advantages include oral administration, low toxicity, and significant clinical and radiologic benefit.
Role in therapeutic strategy	Probenecid may serve as an effective adjunct therapy for calcinosis in JDM, especially in patients with disrupted phosphate metabolism. It offers a non-immunosuppressive alternative when standard treatments fail [33].
Additional notes/comments	The benefit of probenecid appears closely tied to its effects on phosphorus metabolism. Given the limited number of studies, its use should be approached individually and confirmed through further controlled trials.
Level of evidence	Very low (exclusively case reports).
Type of calcinosis treated	Extensive dystrophic calcinosis (JDM), subcutaneous and intermuscular calcinosis and on calcinosis cutis universalis (single JDM case) by Eddy et al. 1997 [83].

**Table A8:** Intravenous Immunoglobulin (IVIG).

Therapeutic class	Intravenous immunoglobulin (IVIG) is an immunomodulatory agent composed of pooled antibodies that support adaptive immunity.
Mechanism of action	IVIG exerts its effects primarily through anti-inflammatory pathways, including suppression of macrophage activation, modulation of inflammatory cytokine release, interference with Fc receptor binding, immune complex neutralization, and complement inhibition [36,37].
Specific indications	IVIG has been used as a therapeutic option for dystrophic calcinosis cutis, calcinosis universalis and ulcerated calcinosis, particularly in autoimmune diseases where conventional treatments have failed.
Dosage and route of administration	IVIG was administered at a dose of 2 g/kg per month in most cases, with variations in infusion schedules. Schanz et al. gave 2 g/kg over 4 days monthly, while Touimy et al. used the same dose monthly without specifying the schedule. Shahani administered 1 g/kg/day for 2 days each month with IV methylprednisolone. Peñate et al. delivered 2 g/kg per month as 0.4 g/kg/day over 5 days. Kalajian et al. used 2 g/kg monthly over several years [36,37,38,39,40].
Clinical efficacy	IVIG has shown variable clinical efficacy in treating calcinosis cutis. Schanz et al. reported significant improvement in a patient with CREST syndrome after five monthly infusions, with resolution of inflammation and restoration of hand function, although some lesions reappeared months later [36]. Touimy et al. described a sustained response in juvenile dermatomyositis with calcinosis universalis, where IVIG led to marked regression of lesions and long-term remission [37]. Shahani observed effective prevention of recurrent calcinosis in a dermatomyositis patient using IVIG with corticosteroids [40]. Similarly, Peñate et al. noted healing of painful ulcers and clinical stability over five years in a case of amyopathic dermatomyositis [38]. In contrast, Kalajian et al. found no benefit in two patients with long-standing disease, indicating that IVIG’s efficacy may be limited in chronic or advanced calcinosis [39].
Side effects and tolerance	Well tolerated in most cases with no adverse effects reported. Mild post-infusion headaches noted in one case, otherwise well tolerated [38].
Advantages/disadvantages	The use of IVIG demonstrated long-term efficacy in select cases, with steroid-sparing effects [37,38]. A key limitation of IVIG therapy is its high cost and the potential need for long-term or indefinite treatment, with variable efficacy observed across cases, particularly in chronic or advanced disease [38,40].
Role in therapeutic strategy	IVIG plays a selective role in the treatment of calcinosis cutis, mainly as a second-line or rescue therapy in severe, refractory, or steroid-resistant cases. It has shown benefit after failure of conventional treatments, including immunosuppressants, and in some cases provided long-term stability with maintenance dosing. However, its effectiveness is inconsistent, as seen in patients with long-standing disease, and it should be considered on a case-by-case basis.
Level of evidence	Very low (case reports and very small case series only).
Type of calcinosis treated	Reported on Calcinosis cutis universalis (JDM) by Touimy et al., localized dystrophic calcinosis (CREST, DM) and ulcerated calcinosis.

**Table A9:** Intralesional corticosteroids.

Therapeutic class	Synthetic analogs of natural steroid hormones produced by the adrenal cortex with immunosuppressive and anti-inflammatory effects.
Mechanism of action	Their benefit in calcinosis cutis is likely due to suppression of local inflammatory responses and inhibition of fibroblast activity, which may promote resorption of calcium deposits [8].
Specific indications	Used in localized calcinosis cutis, particularly when lesions are superficial, painful, or ulcerated, and unresponsive to systemic treatments.
Dosage and route of administration	In Al-Mayouf et al., Depo-Medrol 80 mg and lidocaine were injected under ultrasound guidance using the Barbotage technique [41].
Clinical efficacy	The treated areas showed almost complete ulcer healing, pain relief, and skin softening, despite new lesions developing elsewhere. In Al-Mayouf et al., a 10½-year-old boy with juvenile dermatomyositis and localized calcinosis received injections around the elbow. The treatment led to resolution of calcinosis, improved joint mobility, and no recurrence over two years [41].
Side effects and tolerance	Corticosteroids intralesional injections are generally well tolerated. However, Bonar and Baden reported a rare case where triamcinolone hexacetonide led to the development of calcinosis cutis, highlighting the potential for paradoxical effects in some contexts [42].
Advantages/disadvantages	Intralesional corticosteroids offer localized anti-inflammatory effects with minimal systemic exposure. Their use is limited to accessible lesions and may require repeated sessions.
Role in therapeutic strategy	They may serve as a localized, non-surgical option for reducing inflammation and softening calcific deposits, particularly in cases unresponsive to systemic therapy.
Level of evidence	Very low (single therapeutic case report + safety case reports).
Type of calcinosis treated	Localized, superficial dystrophic calcinosis, peri-articular lesions and on juvenile dermatomyositis.

**Table A10:** Sodium thiosulfate (STS).

Therapeutic class	Sodium thiosulfate (STS) is an inorganic salt historically used intravenously for cyanide poisoning and calcium urolithiasis, and more recently for treating calciphylaxis and tumoral calcinosis [8].
Mechanism of action	Its mechanism of action is primarily attributed to its calcium-chelating properties, forming soluble calcium thiosulfate complexes that enhance the dissolution and clearance of calcium deposits. It also has antioxidant activity that may help reduce local inflammation and pain [8,21].
Specific indications	STS has been indicated in a range of calcinosis cutis presentations, especially localized, symptomatic, or refractory lesions. Topical formulations have proven effective in dystrophic calcification associated with autoimmune diseases and familial tumoral calcinosis, often used as first-line or adjunct monotherapy. Intralesional injections have shown mixed results in early pilot studies, while intravenous STS has been explored in more severe, generalized cases but with disappointing outcomes [48,84,85,86].
Dosage and route of administration	Topical treatment with STS typically involves applying a 10–25% concentration twice daily [46,63]. For more targeted therapy, intradermal STS injections may be administered once every three weeks, using undiluted STS at a concentration of 250 mg/mL in volumes ranging from 0.1 to 1 mL [47].
Clinical efficacy	Wolf et al. reported successful use of topical STS in treating dystrophic and familial calcinosis, with pain relief, reduced calcium deposits, and ulcer healing [86]. In a case series by Ma et al., 68% of autoimmune calcinosis patients improved, with 11% achieving complete resolution [45]. Ricardo et al. reported 78% improvement (20% complete) across 45 patients, with good tolerance [46]. Howard and Smith found STS most effective for small lesions (<2 cm), with no benefit in larger ones [47]. Winter et al. saw limited efficacy of intralesional STS in a small trial, and Song et al. reported no improvement and notable side effects from IV STS in advanced cases [48,85]. These findings suggest that topical and intradermal STS are most effective for small, localized lesions, whereas intravenous STS shows limited efficacy in advanced or widespread calcinosis, particularly in late-stage disease.
Side effects and tolerance	STS was generally well tolerated, especially with topical or intralesional use, where only mild local reactions like irritation or pain were reported. In contrast, intravenous STS was linked to more frequent systemic side effects, including nausea, fatigue, and IV-related complications [48]. Rare adverse events (<2%) included metallic taste, periorbital tingling, hypotension, and transient hearing loss [22].
Role in therapeutic strategy	STS has shown the greatest benefit when applied topically, especially in localized cases. Intralesional STS may help small lesions, while intravenous use is less effective and linked to more side effects.
Level of evidence	Low (retrospective series + one pilot RCT + multiple case reports).
Type of calcinosis treated	Localized dystrophic calcinosis cutis, small lesions (<2 cm), autoimmune CTD-associated calcinosis and familial tumoral calcinosis (topical only).

**Table A11:** Colchicine.

Therapeutic class	Colchicine is an antigout agent.
Mechanism of action	Colchicine interferes with the microtubules of the mitotic spindle and possesses anti-inflammatory properties [59].
Specific indications	In a case report from Vereecken et al., colchicine was used to treat ulcerated dystrophic calcinosis cutis associated with localized linear scleroderma [49]. Colchicine has demonstrated anti-inflammatory effects in calcinosis and is primarily used to manage local inflammation associated with the condition [44].
Dosage and route of administration	Colchicine can be administered orally at a dose of 1 mg per day [19,49,63].
Clinical efficacy	In the study from Vereecken et al., oral colchicine at 1 mg/day led to complete ulcer healing within four months, although calcified deposits remained unchanged [49].
Side effects and tolerance	In renal impairment, colchicine requires dose adjustment and monitoring because of potential neuromuscular toxicity, especially with statins or macrolides.
Advantages/disadvantages	Even if it does not always reduce lesion burden, we value its sometimes very significant pain-relieving effect in our daily practice [8].
Role in therapeutic strategy	Colchicine may serve as an adjunctive therapy to reduce inflammation and promote healing in ulcerated calcinosis when other treatments are limited [49].
Additional notes/comments	To date, only a single case report has documented the use of colchicine in the treatment of calcinosis cutis.
Level of evidence	Very low (single therapeutic case report only).
Type of calcinosis treated	Ulcerated dystrophic calcinosis cutis, localized disease, scleroderma spectrum (localized linear scleroderma).

**Table A12:** Rituximab.

Therapeutic class	Rituximab is an anti-CD20 monoclonal antibody.
Mechanism of action	Rituximab is an anti-CD20 monoclonal antibody that depletes B cells by inducing cell death via immune mechanisms such as antibody-dependent cell-mediated cytotoxicity, complement-dependent cytotoxicity, and apoptosis. In autoimmune conditions, this depletion reduces pathogenic B-cell activity, diminishes autoantibody production, and disrupts immune dysregulation, mechanisms that likely contribute to its therapeutic effect in calcinosis cutis [54].
Specific indications	Used in juvenile dermatomyositis (JDM) with severe or refractory calcinosis [52]. Also applied in CREST syndrome/systemic sclerosis with calcinosis universalis, particularly when lung involvement coexists [53,54].
Dosage and route of administration	Rituximab is typically given by IV infusion. In JDM, Bader-Meunier et al. used two 750 mg/m^2^ doses two weeks apart [52]. In adults, Daoussis et al. administered two 1000 mg doses spaced two weeks apart, repeated every six months [54].
Clinical efficacy	Rituximab has shown mixed efficacy in treating calcinosis cutis. In JDM, Bader-Meunier et al. reported improvement in calcinosis in only 3 out of 9 patients, with no benefit in the remaining 6 [52]. In contrast, Daoussis et al. and Paula et al. described significant regression of calcinosis in patients with systemic sclerosis/CREST after rituximab therapy originally intended for lung involvement [53,54].
Side effects and tolerance	Rituximab was generally well tolerated. However, in the Bader-Meunier study, two patients experienced mild infections at calcinosis sites [52].
Advantages/disadvantages	Rituximab offers a potential therapeutic option for refractory calcinosis, particularly in systemic autoimmune diseases, and may improve both calcinosis and systemic inflammation. Disadvantages include high cost, IV administration, and limited efficacy in some patient subgroups.
Role in therapeutic strategy	Rituximab is not a first-line treatment for calcinosis cutis but may be considered in severe or refractory cases, especially when used for concomitant autoimmune disease activity.
Level of evidence	Low (registry-based case series + multiple case reports).
Type of calcinosis treated	Calcinosis cutis universalis (systemic sclerosis/CREST) and extensive dystrophic calcinosis (subset of JDM).

**Table A13:** Infliximab.

Therapeutic class	Infliximab is a chimeric monoclonal antibody that combines human and mouse components to block tumor necrosis factor-alpha (TNF-α).
Mechanism of action	High levels of TNF-α are often found in JDM, especially in cases with long-standing disease and calcinosis. Infliximab works by binding to and neutralizing TNF-α, thereby reducing chronic inflammation and immune-driven tissue damage—mechanisms particularly relevant in calcinosis linked to prolonged JDM activity [54,55].
Specific indications	Used for refractory JDM with calcinosis, especially when conventional immunosuppressive therapies fail [55,56]. In the article by Tosounidou et al., infliximab was indicated for a patient with refractory calcinosis in the context of an overlap syndrome involving limited systemic sclerosis and myositis [87,88].
Dosage and route of administration	Infliximab has been used both locally and systemically to manage refractory calcinosis in juvenile dermatomyositis. Shiari et al. administered 25 mg of infliximab intralesionally once weekly for six weeks, directly targeting persistent calcified lesions [56]. In contrast, Riley et al. used a systemic approach, giving 6 mg/kg intravenously every four weeks [55].
Clinical efficacy	Improvement in all 5 patients in Riley et al. with better muscle strength, reduced calcinosis and skin signs [55]. Shiari et al. showed size reduction in all 5 patients, in all lesions with intralesional infliximab over 16 weeks [56]. Tosounidou et al. reported sustained improvement of refractory calcinosis, including pain relief and ulcer healing, over 41 months of infliximab treatment in a patient with overlap syndrome [88].
Side effects and tolerance	No major systemic side effects reported. One infected calcinotic abscess resolved with antibiotics in Riley et al. [55]. No side effects reported in intralesional trial [56].
Advantages/disadvantages	Infliximab offers advantages in refractory cases where conventional therapies have failed. It has demonstrated clinical benefit in both systemic and intralesional administration. However, its use is limited by notable disadvantages, including high cost, the need for intravenous administration in a clinical setting, and prolonged treatment duration to achieve sustained results.
Role in therapeutic strategy	Early and aggressive treatment of JDM has been associated with a lower incidence of calcinosis [55]. Infliximab is considered in cases of severe or refractory calcinosis that do not respond to standard therapies.
Level of evidence	Low (small case series + one small prospective trial + case report).
Type of calcinosis treated	Extensive dystrophic calcinosis (JDM), localized, persistent calcinosis lesions, refractory calcinosis in overlap syndromes.

**Table A14:** Thalidomide.

Therapeutic class	Thalidomide is an immunomodulatory agent.
Mechanism of action	Thalidomide reduces TNF-α and IL-6 production by selectively inhibiting their mRNA expression in peripheral blood mononuclear cells. In calcinosis, it is used for its immunomodulatory properties similar to those of infliximab, targeting inflammation and cytokine-driven tissue damage.
Specific indications	Miyamae et al. [57] reported the use of thalidomide in a 14-year-old girl with severe, treatment-refractory juvenile dermatomyositis complicated by inflammatory calcinosis unresponsive to corticosteroids, immunosuppressants, and biologic agents.
Dosage and route of administration	Orally administered at 50 mg/day (1.3 mg/kg/day) for 4 weeks, increased to 75 mg/day thereafter [57].
Clinical efficacy	Led to major clinical improvement, resolution of inflammation, pain, and fever over 18 months; calcinosis remained but was no longer inflammatory or painful [57].
Side effects and tolerance	No side effects were reported in this case.
Role in therapeutic strategy	May be considered a rescue therapy for inflammatory calcinosis when standard treatments and biologics (e.g., infliximab, etanercept) fail [57].
Additional notes/comments	This is the only reported case in the literature; thalidomide use was approved by an ethics committee and administered under close clinical monitoring [57].
Level of evidence	Very low (single therapeutic case report).
Type of calcinosis treated	Inflammatory calcinosis associated with JDM, on a painful, cytokine-driven disease activity.

**Table A15:** JAK inhibitors.

Therapeutic class	Targeted small molecule immunomodulators (e.g., tofacitinib = JAK1/3; ruxolitinib = JAK1/2; baricitinib = JAK1/2).
Mechanism of action	Inhibits JAK–STAT signaling and downstream cytokine/interferon-driven inflammation. In dermatomyositis, JAK/STAT signaling is discussed as potentially relevant both to disease inflammation and calcification pathways (including proposed links to mitochondrial calcium handling) [58].
Specific indications	Most reports are in dermatomyositis (DM) and juvenile dermatomyositis (JDM) with refractory disease and calcinosis cutis (including extensive, progressive calcifications) [58,59].
Dosage and route of administration	Tofacitinib: oral 5 mg twice daily in 2 adults with DM and extensive calcifications (one on monotherapy; one with MTX + low-dose prednisone) [58] Ruxolitinib: oral 5 mg twice daily (2 × 5 mg/day) added in a child with severe MDA5 + JDM; used alongside glucocorticoids/IVIG/hydroxychloroquine in that case [59]. Baricitinib: oral 2 mg/day in a JDM child as part of a multi-therapy regimen (after high interferon signature and lack of improvement) [60].
Clinical efficacy	Tofacitinib (2-case report, adult DM): “fast and persistent” response; no new calcifications over 28 weeks, with existing calcifications regressive or stable; some lesions reportedly shrank and some disappeared by ~28 weeks; functional scores improved [58]. Ruxolitinib (single case, pediatric MDA5 + JDM): skin lesions resolved and no clinical signs of calcinosis remained in the elbow region during follow-up in that report [59]. Baricitinib (single case, pediatric JDM): after adding baricitinib within a combined regimen, calcium deposits progressively softened and function improved over months; no adverse events reported in that letter (but multiple concomitant therapies limit attribution).
Side effects and tolerance	Tofacitinib: generally well tolerated in the 2-case DM report; noted weight gain and a transient hypercalcemia in one patient; otherwise no major adverse events reported there [58]. Ruxolitinib/Baricitinib: the cited case reports describe good tolerance in those individual patients, but emphasize off-label use and need for individualized risk assessment [59,60].
Role in therapeutic strategy	Considered off-label “advanced/targeted” option mainly for refractory DM/JDM (often interferon-high phenotypes) where conventional immunosuppression has failed or complications are severe. Evidence is encouraging but still case based.
Level of evidence	Very low (case reports/very small case series).
Type of calcinosis treated	Mainly dystrophic calcinosis in DM/JDM: includes extensive, rapidly progressive calcifications (multifocal subcutaneous/muscle/skin) and more localized calcinosis cutis (e.g., hands or elbow region).
